# Contraceptive utilization and associated factors among women of reproductive age group in Southern Nations Nationalities and Peoples’ Region, Ethiopia: cross-sectional survey, mixed-methods

**DOI:** 10.1186/s40834-016-0036-z

**Published:** 2017-02-02

**Authors:** Misganu Endriyas, Akine Eshete, Emebet Mekonnen, Tebeje Misganaw, Mekonnen Shiferaw, Sinafikish Ayele

**Affiliations:** 1 Health Research and Technology Transfer Support Process, SNNPR Health Bureau, Hawassa, Ethiopia; 2 Department of Public Health, College of Health Sciences and Medicine, Dilla University, Dilla, Ethiopia

**Keywords:** Contraceptive utilization, Reproductive age women, SNNPR

## Abstract

**Background:**

Though contraceptive utilization has comprehensive benefit for women, it was one of underutilized public intervention in Ethiopia and in the study area. Thus, assessing status and factors affecting contraceptive utilization among women of reproductive age group was found key step for program improvement.

**Methods:**

Community based cross-sectional study was conducted from March to April, 2015 in Southern Nations and Nationalities Peoples’ Region, Ethiopia. A multistage stratified cluster sampling method was used to select 3205 study subjects. Study used both quantitative and qualitative methods. Statistical Package for Social Sciences version 20 was used to analyze quantitative data. The association between variables was determined using odds ratio at 95% confidence interval.

**Results:**

Contraceptive utilization was 53.3% among women of reproductive age groups. Nearly three fourth, (73.6%), of current users were using short-term contraceptive methods. Factors associated with contraception utilization were overall knowledge of and attitude towards contraceptives, age, residence, number of alive children, experience of child death, marital status and deciding number of children. Contraceptive utilization was also affected by various misconceptions.

**Conclusion:**

Contraceptive utilization was below national Health Sector Development Program IV target. Program implementers need to address socio-cultural barriers. Gender myths and specific roles and power inequalities that can function as a barrier to contraceptive utilization should be assessed.

## Background

Contraceptive utilization has multiple benefits to women who are using and community in advance. Contraceptives prevent unintended pregnancies, reduce the number of abortions, and lower the incidence of death and disability related to complications of pregnancy and childbirth. The long-term benefits range from increased education for women and better child health to greater family savings and stronger national economies. Increased contraceptive use and reduced unmet need for contraception are central to improving maternal health, reducing child mortality and combating HIV/AIDS [1–3].

An estimated 225 million women in developing countries would like to delay or stop childbearing but are not using any method of contraception [1]. The worldwide rate of unintended pregnancy in 2012 was 53 per 1,000 women aged 15–44 with the highest regional rate in Africa (80) [4]. Avoiding barriers to the use of contraceptive methods could avert 54 million unintended pregnancies, 79,000 maternal deaths and one million infant deaths each year [5]. Even though remarkable drop in maternal mortality was registered in Ethiopia, the country was still one of ten countries that accounted for nearly 59% of global maternal deaths in 2015 [6].

Different scholars argued that socioeconomic, demographic and psychosocial factors influence contraceptive utilization [7–13]. These factors include women’s perception that they were not at risk of pregnancy, lack of sufficient knowledge, religious or cultural reasons, culturally based gender inequalities, women’s previous experience of child death [14]. Women’s’ and/or couples’ fertility preferences, counseling about family planning methods by health workers [13, 15] and personal income or wealth were also considered factor [13].

The Ethiopian Ministry of Health planned to increase contraceptive prevalence to 66% by 2015 [16]. Nationally, contraceptive prevalence rate (CPR) slightly increased from 29% in 2011 to 42% in 2014 among married women while it increased from 34% in 2011 and 39.8% in 2014 in Southern Nations and Nationalities People Region (SNNPR) [15, 17].

Even though contraceptive methods are made accessible near to household level through health extension program in the country as well as in study area, it was one of underutilized public intervention. Thus, assessing status and individual characteristics affecting contraceptive utilization was found key step for program improvement.

## Methods

Community based cross-sectional study was conducted in SNNPR in 2015. SNNPR is the third largest administrative region of Ethiopia representing about 20% of the country’s population. It is the most diverse region in the country in terms of language, culture and ethnic background. About 93% of the populations are rural. Administratively, the region is divided into 14 zones, 1 city administration and 4 special woredas (districts) [18].

Multistage stratified cluster sampling technique was used. Initially, the region was stratified into three strata: urban, pastoralist and agrarian weredas (administrative structure of about 100,000 population). Using simple random sampling, 20% woredas from each stratum (a total of 40 woredas) were selected. In the second stage, two kebeles (the smallest administrative structure) from each woreda were selected. Finally, two limatbudin (development network of 25–30 households) were selected from each kebele. For all levels of selections, simple random sampling was used as there were lists of structures (woredas, kebeles and limatbudin). All eligible, a total of 3205 non-pregnant women in reproductive age group (15–49 years), in selected limatbudin were included.

Five focus group discussions (FGDs), 10 discussants in each, with women in reproductive age group were conducted. Selection of participants was based on their experience about community practice. They were leaders of one-to-five community development network and lead at least five households for more than two years. The discussion focused on their view on contraceptive methods, methods that most people choose and do not prefer and reason for preference or not, fear of side effects and community acceptance of contraceptive methods.

Ten in-depth interviews (IDIs) were also done; five IDIs with health extension workers/community level service providers/and 5 with woreda program officers. Selection for IDIs was based on experience (above two years). The discussion focused on methods that most people choose and do not prefer and reason for preference or not, fear of side effects and community acceptance of contraceptive methods. All FGDs and IDIs were done in areas selected for quantitative data. FGDs, IDIs with community health worker and woreda health officers covered three agrarian, one urban and one pastoralist woreda. Qualitative data were collected by principal investigators.

Questionnaire was adapted from standard Demographic and Health Surveys (DHS). Questionnaire was prepared in English and translated into Amharic. It was pre-tested and relevant modifications were made. Twenty diploma holder nurses conducted an interview.

Contraception utilization was assessed by asking women for the use of any contraceptive method at the time of data collection. Knowledge of contraceptive was measured using different questions about contraceptives. These were having information on different contraceptives methods, place where a person can get contraceptive, importance and side effects of contraceptives. Having correct answers for at least 75% was considered as good knowledge.

Similarly, attitude was assessed by Likert’s scale. Responses were dichotomized and summed. Having a positive response to at least 75% of statements was considered positive attitude. Attitude statements included feeling towards benefits of contraception (for both woman and family) and side effects.

Data entry and cleaning was done by using Epi Info vision 7 while analysis was done by using IBM SPSS V-20 for windows. Descriptive statistics was used to describe the socio-demographic and other study variables. Bivariate and multivariate logistic regression analysis were used to identify factors associated with contraceptive utilization. Qualitative data were translated and transcribed to English and categorized accordingly to main thematic areas manually.

Ethical clearance was obtained from the Ethical Review Committee of Regional Health Bureau. Informed verbal consent was obtained from each respondent. The information obtained was anonymous and kept confidential.

## Results

### Socio-demographic characteristics of respondents

A total of 3205 non-pregnant women in the reproductive age group (15–49) were included. The mean age of the respondent was 26.25 ± 7.3 years. About half, 1668 (52%), of respondents were protestants and about one forth, 748 (23.3%), were in the age range between 25 and 29 years and more than half, 1810 (56.5%), of respondents were housewives (Table 1).

**Table 1:** Socio-demographic characteristics of respondents in SNNPR, Ethiopia, 2015

Variable (*n* = 3205)	Number	Percent (%)
Age		
15–19 years	658	20.5
20–24 years	532	16.6
25–29 years	748	23.3
30–34 years	508	15.8
35–39 years	390	12.2
40–44 years	130	4.1
45–49 years	25	0.8
I don’t know	214	6.7
Type of residence		
Urban	452	14.1
Rural agrarian	2677	83.5
Rural pastoralist	76	2.4
Educational status		
Illiterate	1201	37.5
Read and write only	177	5.5
Primary school (1–8)	1180	36.8
High school (9–12)	456	14.2
Certificate (10 + 1 and 12 + 1)	94	3.0
Diploma and above	97	3.0
Religion		
Orthodox	1030	32.1
Protestant	1668	52.0
Catholic	87	2.7
Muslim	372	11.6
Pagan/no-religion/	40	1.3
Others	8	0.3
Occupation status		
Housewife	1810	56.5
Government Employ	130	4.1
Private Employ	58	1.8
Agrarian/farmer	273	8.5
Pastoralist	56	1.7
Merchant	306	9.6
Unemployed	87	2.7
Student	481	15.0
Others (Daily laborer, Driver)	4	0.1

### Reproductive history of respondents

About three forth, 2376 (74.1%), were married and from those who were married, about half, 1360 (53.8%), married before age of 18 years (Table 2).

**Table 2:** Reproductive history of respondents in SNNPR, Ethiopia, 2015

Variable	Frequency	Percent
Marital Status (*n* = 3205)		
Married and live together	2376	74.1
Divorced	54	1.7
Widowed	71	2.2
Separated	25	0.8
Single	679	21.2
Wife age at marriage (*n* = 2526)		
Less than 18 years	1360	53.8
18–20 years	496	19.6
Greater than 20 years	343	13.6
I don’t know	327	12.9
Number of alive children (*n* = 3205)		
No child	836	26.1
1–2 children	842	26.3
3–4 children	751	23.4
5 and above children	776	24.2
Number of child death (n = 3205)		
No child death	2767	86.3
1–2 Children	381	11.9
3–4 Children	48	1.5
5 and above children	9	0.3
Woman’s desired number of children (*n* = 3205)		
Not decided	1511	47.1
1–4 children	1001	31.2
5 and above children	693	21.6

### Knowledge of and attitude towards contraceptive methods

**Fig. 1:**
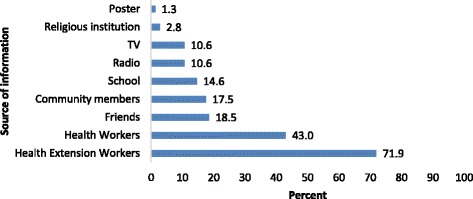
Distribution of Sources of information for contraceptive methods, SNNPR, Ethiopia, 2015

**Fig. 2:**
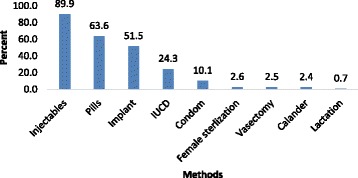
The percentage of women who have knowledge of each method in SNNPR, Ethiopia, 2015

The majority of participants, 2757 (86.0%), heard about at least one type of contraceptive method. Health extension workers were the leading source of information reaching 1981 (71.9%) respondents. The most reported contraceptive methods were injectable, 2479 (89.9%) and pills 1754 (63.6%) (Figs. 1 and 2).

According to methods used to measure knowledge and attitude, 2666 (83.2%) and 2146 (67.0%), of respondents had good knowledge and positive attitude respectively.

### Contraception utilization

Contraceptive utilization was 1708 (53.3%) among eligible women. The use of modern contraceptives (all methods listed except lactation and periodic abstinence) was 1701 (99.6%). Nearly three fourth, 1263 (73.9%) of current users were using short-term contraceptive methods (methods acting for three months or less) from which two women used calendar with pills or condom (Table 3). The percentage of utilization was higher in urban, 247 (54.6%), and agrarian, 1451 (54.2%), as compared to pastoralist area 10 (13.2%).

**Table 3:** Percentage of contraceptive methods used by respondents in SNNPR, Ethiopia, 2015

Contraceptive methods used (*n* = 1708)	Number	Percent
Injectable	1181	69.1
Implant	378	22.1
Pill	70	4.1
IUCD	65	3.8
Rhythm/Periodic Abstinence	6	0.4
Condom	5	0.3
Female sterilization	2	0.1
Lactation	1	0.1

### Reasons for discontinuation of contraceptive use

**Fig. 3:**
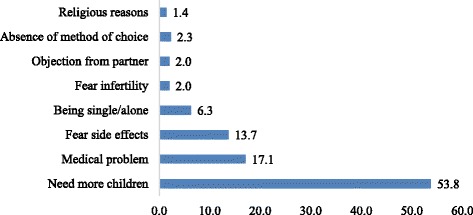
Reasons for discontinuation of contraceptive use among respondents in SNNPR, Ethiopia, 2015

From total respondents, 351 (10.9%) had discontinued using contraceptive methods. Need for more children was the leading reason for discontinuation, 189 (53.8%) while 17.1% and 13.7% add medical problem and fear of side effect as the reason for discontinuation respectively (Fig. 3).

### Reasons for non-use of contraceptive methods

**Fig. 4:**
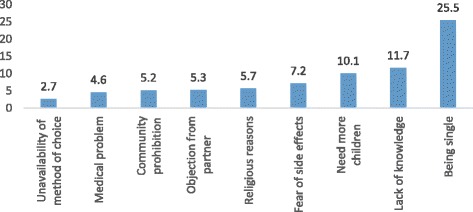
Reasons for non-use of contraceptives among respondents in SNNPR, Ethiopia, 2015

Among women who never used contraceptive method, 292 (25.5%) mentioned being single as main reason for non-use. Need for more children (10.1%), lack of knowledge (11.7%) and fear of side effect (7.2%) were the other reasons given for non-use (Fig. 4).

### Factors associated with contraceptive utilization

In multivariate analysis at 95% CI (*p* < 0.05), variables associated with contraceptive utilization were place of residence, age, religion, marital status, number of alive children, child death experience, deciding number of children, overall knowledge of and attitude to contraceptives (Table 4).

**Table 4:** Bivariate and multivariate analyses of factors associated with contraceptive utilization among women of reproductive age group in SNNPR, Ethiopia, 2015

Variables	Contraceptive utilization		COR (95.0% CI)	AOR (95.0% CI)
	No	Yes		
Residence of the respondents				
Agrarian	205	247	1	
Urban	1226	1451	1.02 [0.83–1.24]	
Pastoralist	66	10	0.13 [0.07–0.25]	0.33 [0.14–0.84]
Educational status				
No formal education	553	825	1	
Primary school	571	609	0.71 [0.61–0.84]	
High school	281	175	0.42 [0.34–0.52]	
Certificate and above	92	99	0.72 [0.53–0.98]	
Age of the respondents				
15–19 years	563	95	1	
20–24 years	198	334	9.99 [7.56–13.22]	
25–29 years	219	529	14.32 [10.95–18.72]	
30–34 years	160	348	12.89 [9.67–17.17]	
35–39 years	147	243	9.79 [7.27–13.21]	0.52 [0.31–0.89]
40–44 years	72	58	4.77 [3.17–7.18]	0.29 [0.15–0.54]
45–49 years	17	8	2.78 [1.17–6.64]	0.29 [0.09–0.86]
I don’t know	121	93	4.56 [3.22–6.44]	0.52 [0.29–0.94]
Religion				
Orthodox	437	593	1	1
Protestant	817	851	0.77 [0.66–0.89]	
Catholic	56	31	0.41 [0.26–0.64]	0.37 [0.21–0.66]
Muslim	160	212	0.97 [0.77–1.24]	
Other	3	5	1.23 [0.29–5.17]	
Pagan	24	16	0.49 [0.26–0.94]	
Occupation				
House Wife	1810	56.5	1	
Employee	188	5.9	0.53 [0.39–0.72]	
Farmer/agrarian/pastoralist	329	10.3	0.33 [0.26–0.42]	
Merchant/daily laborer/	310	9.7	0.65 [0.51–0.83]	
Unemployed	87	2.7	0.14 [0.08–0.24]	
Student	481	15.0	0.05 [0.04–0.07]	
Marital status				
Married and live together	741	1635	1	1
Single	654	25	0.02 [0.01–0.03]	0.05 [0.03–0.09]
Divorced/widowed/separated	102	48	0.21 [0.15–0.30]	0.24 [0.16–0.37]
Number of alive children				
No child	739	97	1	
1–2	248	594	18.25 [14.09–23.63]	3.07 [2.06–4.58]
3–4	228	523	17.48 [13.44–22.73]	3.74 [2.41–5.82]
5+	282	494	13.35 [10.32–17.26]	4.67 [2.91–7.49]
Child death experience of woman				
No	1303	1464	1	
Yes	194	244	1.12 [0.91–1.37]	0.62 [0.48–0.81]
Overall knowledge on FP				
Poor knowledge	515	24	1	
Good knowledge	982	1684	36.79 [24.26–55.83]	15.51 [9.75–24.68]
Attitude towards contraceptive				
Negative attitude	775	284	1	
Positive attitude	722	1424	5.38 [4.57–6.33]	1.97 [1.57–2.47]
Decided number of children				
No	882	629	1	
Yes	615	1079	2.46 [2.13–2.84]	1.58 [1.29–1.93]

### Qualitative findings

To triangulate quantitative findings, key qualitative results from both FGDs and IDIs were summarized to themes that emerged during discussion. Themes emerged were awareness and utilization, method preferences, fear of side effects and misconceptions.

The following are some of points raised by FGD participants.

*“Some women said that implant moves to other body parts from the insertion site. It interferes with the routine activities. If users of implant develop any headache or abdominal cramp, they associate the situation with family planning method use and don’t prefer long acting contraceptives”.* ***/30, Agrarian FGD participant/***

*“My friend said if the menstruation decreases or disappears, the dirty blood would accumulate and can cause cancer. I think people need more information on side-effects and what they should do when they have those side effects* ***/32, Agrarian FGD participant/***

*“I used injection but immediately I stopped because I heard from my neighboring that the method can cause kidney disease, hypertension and other health disease”.* ***/29, Agrarian FGD participant/***

*“Young women do not use contraceptive before their first pregnancy; because if they use it, their uterus may become dirty and as a result they may not get pregnant”.* ***/33, Agrarian FGD participant/***

*“I do not want to use contraceptive. I want more children. I would be happy if I could give 17 children”.* ***/29, Pastoralist FGD participant/***

The following are key points raised by health extension workers and woreda program officers.

*“When client come to health facility for the first time, different choice of contraceptives were not introduced by service providers and they are not asked for their choice but Depo-Provera is simply given and women continue using it”.* ***/27, urban health extension worker/***

“*Depo-Provera is preferred by young women because it acts only for three month and it is easy to stop without seeking help when they want to be pregnant”* ***/35, Agrarian program officer/***

*“Most clients take contraceptive without the permission from their partner; for this reason, the simplest method preferred by clients is injectable as one cannot detect if a woman is using contraceptive easily”.* ***/29, pastoralist program officer*** */*

“*IUCD is not preferred due to fear of the procedure, belief that it may cause wound around their reproductive organ especially uterus and may disappear after insertion during sexual intercourse”.* ***/38, Agrarian program officer*** */*

*“In our norm, having sex before marriage and using condom are taboo. So, young woman doesn’t use contraceptive. In addition, some educated people who have knowledge about contraceptives do not use contraceptives and tell us that they are using natural methods”.* ***/25, Agrarian health extension worker*** */*

*“In our community older women use contraceptive more than young women. This is because in our culture, women do not want to be pregnant when their daughters marry and become pregnant and in front of son-in-law”.* ***/26, pastoralist health extension worker*** */*

*“For a woman, it is not easy to use contraceptive methods without permission from her husband as she has no power to decide. First we should work on men because it is the one who decides contraceptive utilization; but convincing men is very difficult as our culture accepts many children as prosperity.”* ***/29, pastoralist program officer*** */*

## Discussions

Contraceptive utilization is one of indicators that can inform status of family planning programs. More than half (53.3%) of the study subjects were using any type of contraceptive methods. Although contraception utilization was higher than previously reported in study area [15, 17], it was lower than ministry of health plan that targeted to reach 66% in the year 2015 [19].

Nearly three fourth (73.9%) of current users were using short term contraceptive methods mainly injectable. This finding was consistent with other previous studies in Ethiopia [8, 15, 17, 20–22], Kenya [23] and Ghana [24]. FGD participants also agreed that due to the simplicity of the procedure, popularity by promotion and availability in health facilities, injectable was the most chosen contraceptive method. In addition, in-depth interview with providers also indicated that when clients come to health facility for the first time, some providers simply give injectable without informing choices and women continue using it.

Women in the age range of 35 to 39, 40 to 44 and 45 to 49 years were 52% [AOR 95% CI 0.31–0.89], 29% [AOR 95% CI 0.15–0.54] and 29% [AOR 95% CI 0.09–0.86] less likely to use contraceptive methods as compared those in age range of 15 to 19 years. Previous studies also showed that older age negatively influenced contraceptive use [13, 25].

Avoiding barriers to the use of contraceptive methods could avert globally 54 million unintended pregnancies, 79,000 maternal deaths and one million infant deaths each year [5]. Due to different factors, in 2012, unintended pregnancy in Africa was the highest (80 per 1,000 women aged 15–44) [4]. Women sexually active and living alone are also at risk of unintended pregnancy. Researches from Ethiopia reported that younger age groups (20–24 years) were at risk of unwanted pregnancy [26, 27] while single women were more likely to have induced abortion [27] which is major cause of maternal mortality. In this study, those who were single or not with partner were less likely to utilize contraceptive methods as compared to those women with partner. Those who ever married but not living with partner (divorced, widowed or separated) at time of data collection were 24% less likely to use contraceptive than married and living with partner with AOR at 95% CI 0.24 [0.16–0.37]. In-depth interview with community level health care workers also showed that in some communities, there was community taboo of using contraceptives unless living with partner.

Majority, 88%, of participants heard at least one contraceptive method and awareness of contraception was also high. Having overall good knowledge of and attitude to contraceptives were positively associated with contraceptive use with AOR 15.51 [95% CI 9.75–24.68] and 1.97 [95% CI 1.57–2.47] respectively. Even though the benefits of contraceptive were acknowledged by majority of study participants, lack of knowledge, desire to have many children and fear of perceived side effects of contraceptives were mentioned as reasons for non-use as also reported by other researches [13, 15, 28–32].

Though good level of contraceptive awareness was noted by quantitative interview, FGDs with women showed that contraceptive utilization was affected by various misconceptions. The misconceptions ranged from fear of side effects to association of death of a woman with contraceptive use. Some women believed menstruation as a sign of being healthy and added if menstruation decreases or disappears, the dirty blood would accumulate and can cause cancer. They also believed that young women should not use contraceptive before their first pregnancy; because if they use, their uterus may become dirty and as a result they may not get pregnant. The in-depth interviews findings also indicated that long acting methods particularly implants and IUCD were not preferred by community due to fear of procedure and side effects. Such myths and misconception were also reported in Nigeria [11]. Even though these type of thoughts were from minor groups, they need attention as they have potential to get majorities concern especially in rural communities.

Rural FGDs participants reported that people in the rural areas prefer many children because children are sources of labor for families. But in general sample, women having 1–2 children were 3.07 times more likely to use contraceptives than those who had no children [AOR 95% CI 2.06–4.58] and women having 3–4 and more than five children were 3.74 times more likely [AOR 95% CI 2.41–5.82] and 4.67 times more likely [AOR 95% CI 2.91–7.49] than women who had no children respectively. This was in line with other findings that reported as the number of living children increases, use of contraceptives increases [12, 33]. Pastoralist FGD participants preferred having as many children rather than using contraception. This was enforced by in-depth interview findings; women in pastoralist area have no or limited power to decide and use contraceptive. Women from pastoralist were 33% less likely to use contraceptives than agrarian with AOR 0.33 [95% CI 0.14–0.84].

Improvement of the status of women in the family and in society can contribute to smaller family size, the opportunity for women to plan births and also improves their individual status. Countries are recommended to respect and ensure, regardless of their overall demographic goals, the right of persons to determine, in a free, informed and responsible manner, the number and spacing of their children [34]. But in this study only about half, 52.8%, decided number of children to have. Women who had decided number of children were 58% more likely to utilize contraceptives than those who had not decided with AOR at 95% CI 1.58 [1.29–1.93]. Child death also matters on deciding family size. Women who had experienced child death were 62% less likely to utilize contraceptive than who had not experienced child death. 0.62 [0.48–0.81]. This might be due to an attempt to replace the lost ones and want to have more children.

This study was limited in assessing male partner involvement and support, gender myths and specific roles and power inequalities which can function as a barrier to contraceptive utilization. Results might have been affected by social desirability bias since questionnaires were collected by interviewers. In addition, risk for pregnancy or sexual activity was not assessed which could be reason for non-use of contraceptive/s.

## Conclusions

The contraceptive utilization was below national HSDP IV target. Factors associated with contraceptive utilization were overall knowledge of and attitude towards contraceptive, age, residence, number of alive children, experience of child death, marital status and deciding number of children. In spite of high level of awareness of contraceptive methods, the utilization was affected by various misconceptions, particularly in long acting family planning methods.

Program implementers need to address socio-cultural barriers. Gender myths and specific roles and power inequalities that can function as a barrier to contraceptive utilization should be assessed.

## Abbreviations

AIDS: Acquired immune deficiency syndrome
CPR: Contraceptive prevalence rate
DHS: Demographic and health surveys
HIV: Human immunodeficiency virus
HSDP: Health sector development program
IBM: International Business Machines Corporation
IUCD: Intrauterine contraceptive device
MMR: Maternal mortality ratio
MoH: Ministry of health
SNNPR: Southern Nations, Nationalities, and Peoples’ Region
SPSS: Statistical package for social sciences
